# African Case Studies for Public Health Volume 2

**DOI:** 10.11604/pamj.supp.2018.30.1.16124

**Published:** 2018-05-31

**Authors:** Ghada N Farhat, Sorie Dumbuya, Scott JN McNabb

**Affiliations:** 1 Emory University, Rollins School of Public Health, Atlanta, GA, USA

**Keywords:** Case study, Field epidemiology, Public health

## Editorial

In this supplement of the *Pan African Medical Journal*, we introduce the second series of African case studies for public health. The first series [1] was published in May 2017 and included a set of 11 case studies, all based on real public health events occurring in various African countries and all written by experienced Africa-based public health trainers and practitioners. The current supplement introduces 16 new case studies and expands the portfolio of public health functions, health issues and country-specific settings covered in the prior issue. Together, the two series published thus far address a major gap encountered in public health training programs in Africa – the lack of problem-based learning exercises that are locally relevant to African contexts. To offset this gap, the Rollins School of Public Health, Emory University – in close collaboration with the African Field Epidemiology Network (AFENET) – developed a training curriculum [2] and implementation plan for the design and development of context-specific and culturally tailored public health case studies. The case studies that appear in this issue are the product of two cohorts of trainees who attended the training program at Emory University in 2016 and 2017. Public health functions targeted in this set of case studies include outbreak investigation, research design and secondary data analysis. The range of health issues covered spans anthrax, cholera, measles, rubella, pertussis, meningitis, tuberculosis, schistosomiasis, foodborne illness, paralytic shellfish poisoning, heavy metal poisoning and congenital syphilis. Countries represented include Burkina Faso, Ethiopia, Ghana, Kenya, Namibia, Nigeria, South Africa, Tanzania, Uganda and Zimbabwe. These case studies and future ones are accessible to public health professionals and educators from various universities and Field Epidemiology and Laboratory Training Programs (FELTPs) in Africa and across the world. We trust they will help train the next generation of African disease detectives and public health leaders. This initiative was funded by AFENET. We acknowledge with gratitude the contribution and dedication of the Emory University team (Casey Daniel Hall, Calbeth Alaribe, Hailey McLeod, Evelyn Kamgang and Mikayla Farr) and the AFENET team (Joseph Asamoah Frimpong, Dr. Olivia Namussisi and Dr. Chima Ohuabunwo).

## Competing interest

The authors declare no competing interest.
